# Resolution of chronic inflammatory disease: universal and tissue-specific concepts

**DOI:** 10.1038/s41467-018-05800-6

**Published:** 2018-08-15

**Authors:** Georg Schett, Markus F. Neurath

**Affiliations:** 1 Department of Internal Medicine 3, Friedrich-Alexander University Erlangen-Nürnberg and Universitatsklinikum Erlangen, 91054 Erlangen, Germany; 2 Department of Internal Medicine 1, Friedrich-Alexander University Erlangen-Nürnberg and Universitatsklinikum Erlangen, 91054 Erlangen, Germany

## Abstract

Inflammation and its resolution is under-studied in medicine despite being essential for understanding the development of chronic inflammatory disease. In this review article, we discuss the resolution of inflammation in both a biological and translational context. We introduce the concept of impaired resolution leading to diseases like rheumatoid arthritis, Crohn's disease, and asthma, as well as the cellular and molecular components that contribute to resolution of joint, gut, and lung inflammation, respectively. Finally, we discuss potential intervention strategies for fostering the resolution process, and their implications for the therapy of inflammatory diseases.

## Abstract (web summary)

Inflammation is a component of many chronic inflammatory diseases and yet it is understudied in medicine. Here, the authors review novel insights in to inflammation and how impairment of its resolution can lead to diseases.

## Introduction

Inflammation is characterized by a sequence of events comprising an induction phase, which leads to the peak of inflammation and is gradually followed by a resolution phase. The induction phase of inflammation is designed to allow fast and robust immune activation that is required for effective host defense. It is initiated by the sensing of exogenous and endogenous danger signals resulting from mechanically, chemically, or biologically induced tissue damage followed by the recruitment of effector cells, which orchestrate an inflammatory response characterized by the release of lipid and protein mediators of inflammation^1^.

**Fig. 1:**
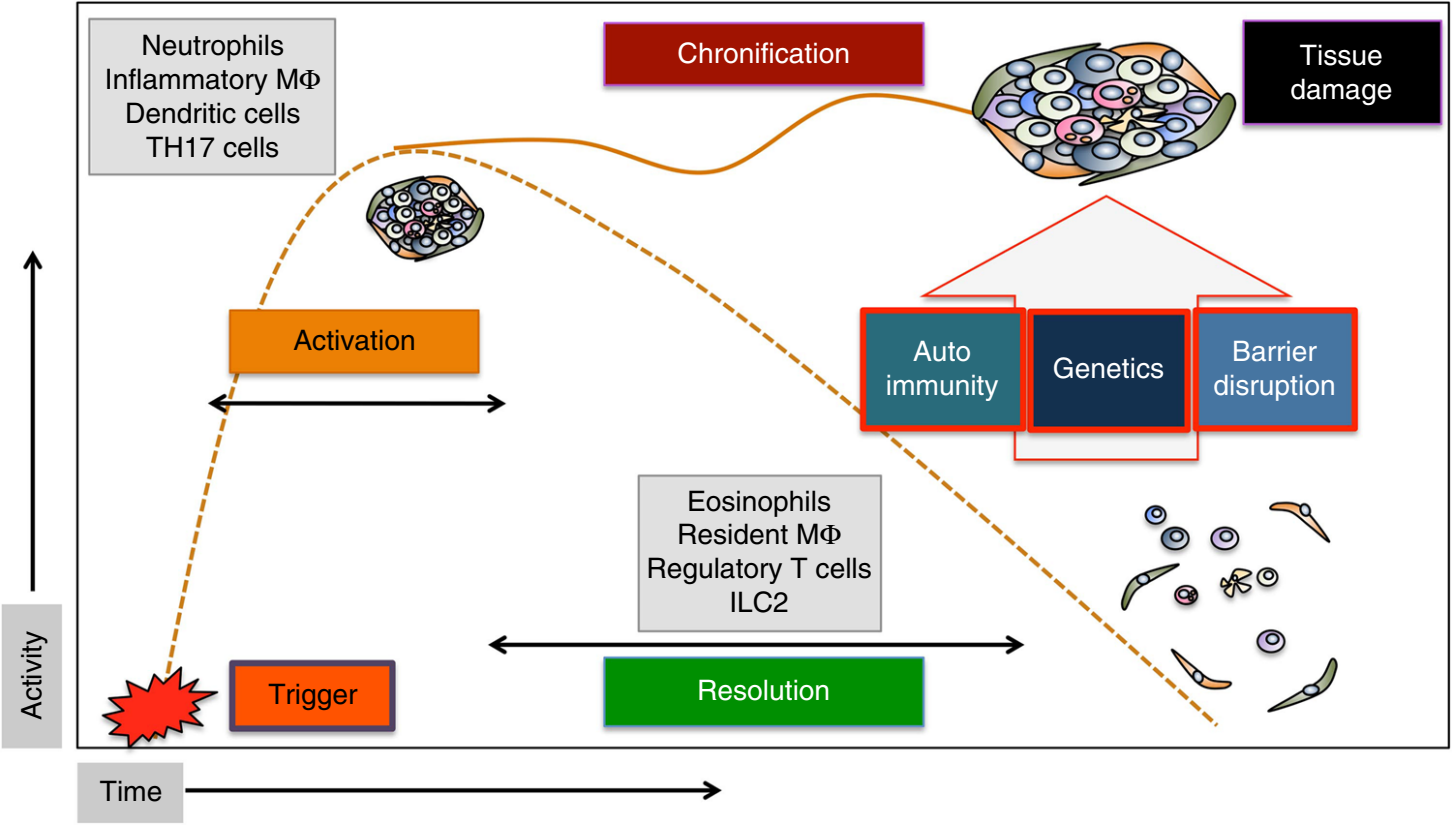
Activation, resolution, and the process leading to chronicity of inflammation. Time course of the inflammatory response with activation phase, peak phase, and resolution phase. Failure of resolution leads to chronic inflammatory diseases. Key cells of the activation and resolution phase are shown in the gray squares. ILC innate lymphoid cells, TH17 T helper cells 17, MΦ macrophages

The resolution phase of inflammation is essential to curtail inflammation and restore tissue homeostasis once the danger signal has been eliminated^2^. It is therefore self-evident that the resolution process is tightly controlled^2^. Failure to resolve inflammation leads to chronic inflammatory diseases at the inner surfaces of the body, such as arthritis, colitis, or asthma, which are associated with irreversible tissue damage and increased risk for development of cardiovascular disease, cancer, and osteoporosis^3–5^. Factors that can overcome effective resolution processes are (i) genetic factors that encode for exaggerated immune responses (e.g., HLA-B27 in arthritis, NOD2, and IL-10R mutations in colitis) or modulate the gut microflora, (ii) mechanisms that promote the development of autoimmunity (e.g., citrullinated protein antibodies in arthritis), and (iii) processes leading to impairment of the barrier function of epithelial surfaces leading to prolonged microbial exposure (e.g., defensin and mucin reduction in inflammatory bowel diseases) (Fig. 1).

Our current concept on the nature of resolution of inflammation is dominated by specific pro-resolving lipid mediators that orchestrate the resolution process. These mediators have been extensively reviewed elsewhere^6^. However, like the induction phase of inflammation depends on both lipid (e.g., prostaglandin E2) and protein (e.g., tumor necrosis factor) mediators, also the resolution of inflammation is likely to be based on a network of lipid and protein mediators rather than being exclusively controlled by lipids. Furthermore, in addition to universal principles underlying resolution of inflammation, disease-specific, and tissue-specific processes may exist that guide resolution in specific inflammatory conditions. This article will first summarize the universal principles of the resolution process before addressing tissue-specific and disease-specific mechanisms functional in arthritis, colitis, and asthma, as paradigm examples for chronic inflammatory diseases^3^.

## Key aspects of resolution of inflammation

Successful resolution of inflammation is based on a number of key events. At the molecular level, pro-inflammatory mediators need to be effectively antagonized and catabolized. At the cellular level, immune cell numbers at inflammatory sites need to decline, and at the macroscopic level tissue integrity has to be restored by the activation of tissue-resident cells that induce repair^7^. To some extent, resolution of inflammation represents a “clean-up” process, which follows the “concert” of inflammation. As such, resolution is a highly robust process, which is difficult to overwrite. In consequence, the vast majority of inflammatory responses occurring in humans are self-limited. Furthermore, at the experimental level it is still challenging to mimic chronic inflammation in animal models as many inflammatory models such as dextran sodium sulfate-induced colitis, antigen-induced arthritis, imiquimod-induced psoriasis, or thioglycolate-induced peritonitis are spurious and spontaneously resolve after several days or weeks of active inflammation^8,9^.

## Universal mechanisms of resolution of inflammation

Three key processes have been identified, which are intrinsic to resolution of inflammation and represent an over-reaching principle of the resolution process, which is functional across different tissues and diseases. These processes are discussed in the following chapter.

### Cessation of neutrophil influx

Neutrophil swarming to sites of damage is the initial step in inflammation^10^. Resolution of inflammation depends on stopping neutrophil recruitment, which is the most abundant leukocyte population at inflammatory sites. This process is in part controlled by pro-resolving lipid mediators (resolvins), reviewed elsewhere^7^ (see also Table 1). Briefly, resolution seems to go along with a class-switch from the production of pro-inflammatory (such as PGE2 and LTB4) to pro-resolving lipid mediators such as prostaglandin D2, 15deoxy-delta-(12-14)-prostaglandin J2, lipoxin A4 (LXA4), resolvin E1 (RvE1), protectin D1, and maresin-1^11^. These mediators can block neutrophil recruitment, partially by downregulating their chemokine receptors such as CXCR2 rendering them unresponsive to neutrophil-activating substances such as leukotriene B4, KC, and complement factor 5^12^.

**Table 1:** Overview of key mediators and processes triggering the resolution of inflammation

Molecules and pathways	Source	Function
Lipid mediators		
Lipoxin A4	MPH and eosinophils	Inhibition of PMN influx, efferocytosis
Resolvin E1	MPH and EC	Inhibition of PMN Influx
Protectin D1	TH2 cells	Inhibition of PMN influx, efferocytosis
Maresin 1	MPH	Inhibition of PMN influx
Cytokines		
IL-4	Eosinophils	AAM differentiation
IL-13	Eosinophils	AAM differentiation
IL-10	AAM and Treg	AAM differentiation
TGF-β	AAM and Treg	Tissue repair and PMN apoptosis
IL-9	ILC2 and TH9 cells	Treg activation
IL-22	ILC3 and TH17 cells	Epithelial barrier function
Metabolic factors		
Adenosin	Many cells	AAM differentiation
Itaconate	Many cells	AAM differentiation
AMPK	Many cells	AAM differentiation
ROS	Neutrophils	NETosis
Other proteins		
Netrin 1	Neuronal cells	AAM differentiation
TRAIL	T cells	PMN apoptosis
FASL	Multiple cells	PMN apoptosis
Annexin 1	PMN and MPH	AAM differentiation, efferocytosis
Other factors		
Vagus nerve stimulation	Acetylcholin (Vagus)	AAM differentiation

AAM alternatively activated macrophages; AMPK adenosine monophosphate-activated protein kinase; EC endothelial cells; FASL Fas ligand; IL interleukin; ILC innate lymphoid cells; MPH macrophages; PMN polymorphonuclear neutrophils; ROS reactive oxygen species; TH17 T helper cells 17; TGF transforming growth factor; TRAIL tumor necrosis factor-related apoptosis inducing ligand

Pro-resolving lipid mediators are produced by a concerted action of arachidonate lipoxygenases (ALOX), which catalyze the production of pro-resolving lipid mediators and are highly expressed in eosinophils, alternatively activated macrophages and certain tissue-resident macrophages, cell types that predominate during the resolution process and contribute to tissue homeostasis^13,14^. Another important feature of pro-resolving lipoxygenases seems to be the oxidation and modification of membrane phospholipids in eosinophils and macrophages, a process that changes biophysical properties of cell membranes and contributes to the non-immunogenic clearance of apoptotic cells and the maintenance of immune tolerance during the resolution process, as well as to cessation of bleeding after injury^14^.

### Neutrophil death and removal

**Fig. 2:**
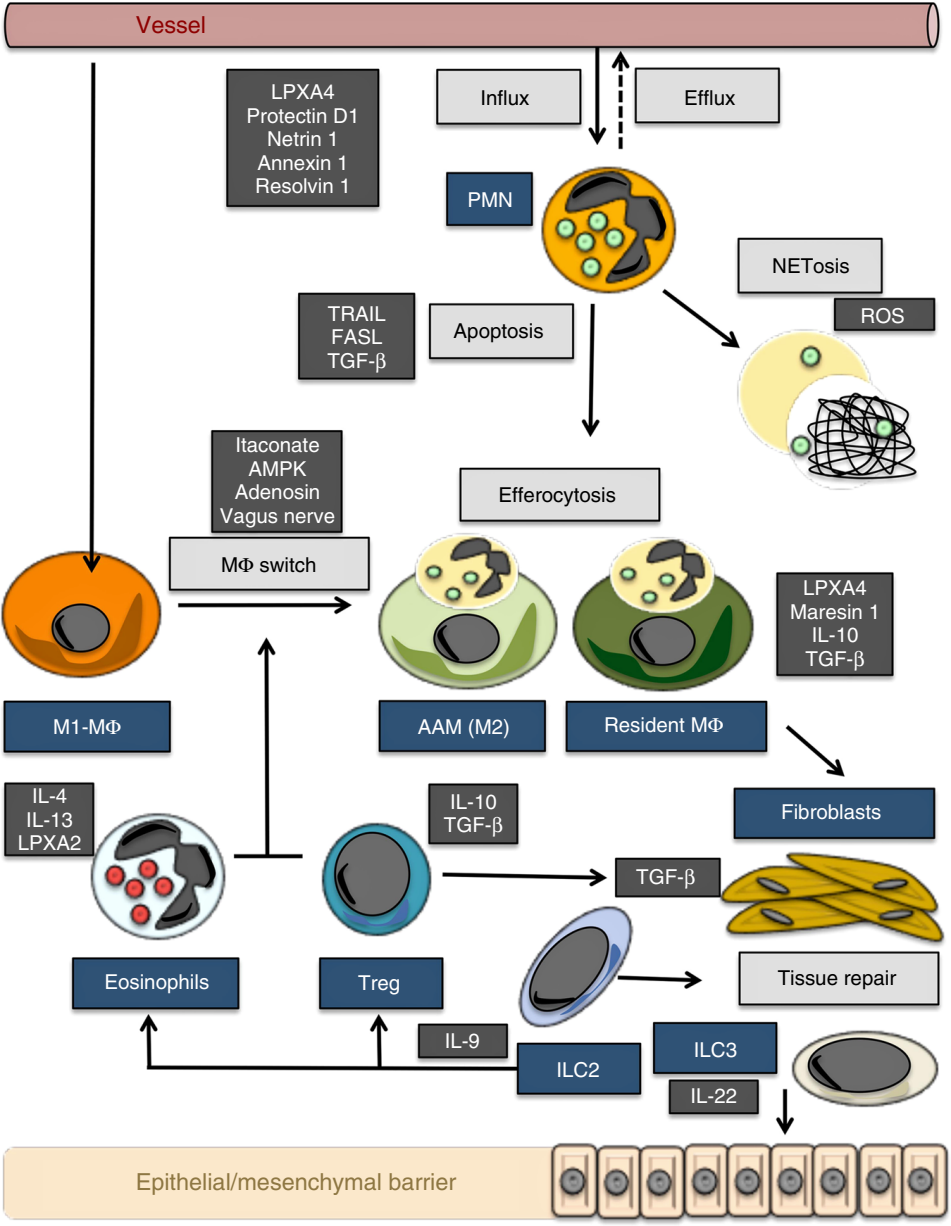
Functional resolution map. Map of resolution with key mechanisms (bright gray squares), cells (blue squares), and molecules (dark gray squares). AAM alternatively activated macrophage, AMPK adenosine monophosphate-activated protein kinase, ILC innate lymphoid cells, IL interleukin, LPX lipoxin, MΦ macrophages, PMN polymorphonuclear neutrophils, TGF transforming growth factor, TRAIL tumor necrosis factor-related apoptosis inducing ligand, FASL Fas ligand, ROS reactive oxygen species

Resolution processes require the removal of tissue neutrophils (Fig. 2), a process that has been recognized more than 100 years ago^15^. Neutrophils are terminally differentiated cells with a short half-life. Hence, the journey of neutrophils from the bone marrow to the sites of inflammation is, for the most part, unidirectional with cells dying in the target tissue. Neutrophil apoptosis can be induced by expression of death ligands such as TRAIL or FasL, produced by macrophages^16,17^, or by transforming growth factor beta (TGFβ), produced by regulatory T cells during the resolution phase of inflammation^18^. Apoptotic neutrophils are rapidly engulfed by macrophages—a process known as efferocytosis. This uptake essentially depends on the expression of “eat-me” and “find-me” signals on apoptotic neutrophils such as phosphatidylserine^19^, which are recognized by specific receptors such as TIM1 and TIM4 on the surface of resident macrophages^20^. Neutrophil death and engulfment by macrophages represents an essentially anti-inflammatory and pro-resolving signal^21^.

### Changes in macrophage function

Monocyte-derived macrophages are key players of the innate immune response, where they contribute to cytokine production and pathogen clearance. Consistently, genome-wide association studies recently identified key driver genes of macrophages for inflammatory disorders^22^. During the course of inflammation, however, monocyte-derived macrophages seem to acquire essential anti-inflammatory and pro-resolving functions^23^. They clear apoptotic cells, start to release pro-resolving lipids^13^, express anti-inflammatory receptors such as TGF-R2 and FPR2, and synthesize increased concentrations of immune regulatory intracellular messengers such as cAMP^24^. These changes appear to be based on both intrinsic and extrinsic mechanisms.

Intrinsic mechanisms triggering the switch in macrophage function: Recent insights into the metabolic control of macrophage function may open new concepts in understanding the switch in macrophage function during resolution. Notably, macrophage function has been associated with different patterns of energy production, which guide the cells functional state. A well-characterized effect of macrophage activation by lipopolysaccharide is the shift from oxidative phosphorylation to aerobic glycolysis, which is also known as the Warburg effect^25^. Cells involved in the activation phase of inflammation, such as inflammatory M1-like macrophages, must provide energy rapidly to fuel inflammation, which is essentially accomplished by glycolysis^26^. At the same time, energy production by oxidative phosphorylation through the Krebs cycle is defective in inflammatory M1 macrophages. This phenomenon has also been described as a “broken” Krebs cycle by O’Neill and colleagues^27^, which is characterized by the accumulation of the metabolites citrate and succinate. Increase of citrate and succinate is the consequence of suppression of the conversion of citrate to a-ketoglutarate as well as oxidation of succinate, respectively. Citrate has proinflammatory functions by facilitating prostaglandin generation^28^. Succinate, on the other hand, acts in proinflammatory manner as well by fostering the release of TNFa and IL-1β, stabilizing hypoxia inducible factor 1-alpha (HIF1α) and supporting the production of reactive oxygen species (ROS)^29–31^.

On the other hand, M2-like macrophages, which are involved in immune regulation and resolution of inflammation, preferentially use fatty acid oxidation through Krebs cycle as a main source for energy production. In the context of metabolic control of resolution of inflammation, the Krebs cycle metabolite itaconate appears to be an attractive mediator. Manipulation of IRG1, the enzyme responsible for itaconate production, has shown to affect the pro-inflammatory function of macrophages, supporting the concept that itaconate induces a regulatory pro-resolving macrophage phenotype^32^. Itaconate thereby acts by modifying proteins via alkylation of cysteine residues. One of the targets, which is modified and inactivated by itaconate, is Kelch-like ECH-associated protein-1, which functions as key repressor of the anti-inflammatory transcription factor Nrf2 in macrophages^33^. Macrophages treated with itaconate show impaired production of key pro-inflammatory cytokines like IL-1β, IL-12, and IL-6^34^. In part, this effect seems to be based on itaconate-mediated suppression of succinate oxidation^35^, which—as described previously—is an central proinflammatory immune metabolic in macrophages. Altogether, these effects render itaconate as crucial anti-inflammatory metabolite that limits inflammation and potentially also fosters resolution of inflammation.

Extrinsic mechanisms triggering the switch in macrophage function: This switch in macrophage function during resolution may be additionally supported by eosinophils^36^ as well as tissue-resident group 2 innate lymphoid cells (ILC2s) that produce IL-4 and IL-13 and trigger a regulatory “M2-like” macrophage phenotype through activation of STAT6^37,38^. Regulatory T cells, producing TGF-β and IL-10, as well as other soluble mediators expressed during the resolution process, such as adenosine, annexin 1, and netrin-1, may further add to this phenotype^39,40^. Newer findings, however, also challenge the concept of a simple switch of monocyte-derived macrophages into a pro-resolving “M2-like” phenotype as the only mechanism involved the resolution of inflammation. Tissue-resident macrophages, that are of embryonic origin and populate virtually all tissues in steady-state conditions, may play a so far underappreciated role in the resolution process^41^. Although phenotypically distinct, many tissue-resident macrophages such as microglia, resident peritoneal macrophages, or alveolar macrophages display an regulatory phenotype that is characterized by the constant production of anti-inflammatory cytokines and lipid mediators, as well as by an increased capacity to clear dying cells in a non-inflammatory fashion^13^.

## Tissue-specific mechanisms of resolution of inflammation

Tissue-specific resolution processes complement the aforementioned universal mechanisms in resolution of inflammation. Since the pathways involved in the initiation of arthritis, inflammatory bowel disease, or asthma differ considerably from each other with respect to cytokine involvement, composition of the resident tissue, and microbial load, it is not surprising that customized resolution processes exist for specific tissues, which are guided by the local microenvironment^3^. This is particularly relevant for tissues that represent inner surfaces of the body that are characterized by specific environmental settings. In the following chapter, we will therefore put a focus on the specific mechanisms of resolution of inflammation in diseases such as arthritis, colitis, and asthma.

### Resolution of arthritis

Rheumatoid arthritis is the most severe inflammatory joint disease exhibiting a remarkable level of chronicity. While pro-inflammatory mediators, like TNFα and IL-6, are well defined in arthritis^4^, pro-resolving cytokines have not been defined until recently. In fact, IL-9 appears to be a key resolution cytokine in arthritis^42^. Antigen-induced arthritis, which represents a self-limited arthritis model, becomes highly chronic in the absence of IL-9, while IL-9 overexpression effectively induces the resolution of arthritis in chronic models. Mechanistically, IL-9 serves as an autocrine growth factor for ILC2s. These cells have pro-resolving properties by activating the suppressive function of regulatory T cells in a cell–cell contact GITRL and ICOSL-dependent fashion. Notably, in the gut and lungs, where IL-9 is preferentially synthesized by T cells, this cytokine fuels rather than resolves inflammation supporting the concept of disease-specific resolution processes^43,44^.

ILC2s appear to be central components of the resolution phase of several forms of arthritis^42,45^. Apart from engaging regulatory T cells, ILC2s control eosinophil homeostasis through production of IL-5^38,46^ and support the differentiation of regulatory macrophages^36–38^. Their pro-resolving and immune regulatory function is also illustrated by the decline of ILC2 numbers in inflammation during obesity, which is associated with resistance to resolution of inflammation and higher chronicity of rheumatoid arthritis^47^. The role of ILC2s in resolution of inflammation in arthritis also sheds light on the function of eosinophils. While in allergic asthma, eosinophils are essentially pro-inflammatory cells, which are targeted by IL-5 neutralization^48^, the same cells play a role in the resolution of inflammation in arthritis^36^. Already, Paul Ehrlich described eosinophilia as the “aurora” of resolution at the dawn of the inflammation process more than 100 years ago^49^. In accordance, lack of eosinophils has been shown to prolong peritonitis^50^, colitis^51^, and arthritis^36^ in mouse models, whereas increasing eosinophil numbers by overexpression of IL-5 strongly inhibits experimental arthritis^36^.

Another important tissue-specific resolution process in arthritis is the formation of aggregated neutrophil extracellular traps (aggNETs). This mechanism is based on neutrophil death by NET formation, where the dying cell expels their DNA into the extracellular space^52^. In case of very high neutrophil densities, like those found in gouty arthritis, NETosis can lead to the formation of aggNETs, large agglomerates of dead neutrophils, which macroscopically appear as tophi. These structures are highly potent to trap and degrade cytokines by released neutrophil proteases cleaving essential pro-inflammatory cytokines and chemokines, like TNF, IL-1, IL-6, and CCL2^53^. AggNET formation thereby removes the “fuel” of inflammation leading to the collapse of the inflammatory process and explaining the rapid cessation of the gout attack. ROS, such as superoxide anions and peroxide, are centrally involved in this aggNET-based resolution process. If ROS production is impaired, e.g., by induced mutation of NOX enzymes in mice or by mutation of the enzyme in human chronic granulomatous disease, NET formation is severely impaired and no proper resolution of inflammation is possible^52^.

Finally, in arthritis, the functional state of resident mesenchymal cells appears to decide upon resolution and chronicity of arthritis. Metabolic changes in synovial fibroblast-like cells, such as the induction glycolysis^54^, as well as expression of molecules, such as FAP, involved in tissue invasiveness of these cells, have been associated with the failure of resolution and the development of chronic arthritis^55^.

### Resolution of colitis

Chronic inflammatory bowel diseases, like Crohn's disease and ulcerative colitis, are severe inflammatory conditions that preferentially affect the distal small intestine or colon with little signs of spontaneous resolution^5^. Resolution of colitis appears to essentially depend on regulatory T cells. It has been shown that during the process of resolution, T cells change their transcriptional profile and acquire regulatory properties with induction of the canonical TGF-beta pathway^18^. Furthermore, IL-10 and IL-35 production by Treg cells supports the resolution process^56,57^. In this context it is particularly remarkable that some of these regulatory T cells originate from the trans-differentiation of TH17 cells^21^. Regulatory T cells have shown to effectively suppress pro-inflammatory immune responses in experimental colitis^58^. For instance, in the adoptive T cell transfer model of colitis, transfer of naturally occurring regulatory T cells and ex vivo TGFβ-induced regulatory T cells permit resolution of colitis^59^. Interestingly, a pilot study in Crohn's disease used ex vivo generated ovalbumin-specific regulatory T cells from the peripheral blood for clinical treatment^60^. The administration of such antigen-specific Treg cells was well tolerated and resulted in dose-dependent efficacy, indicating that Treg-based immunotherapeutic approaches hold promise for resolution of inflammation.

With respect to resolution of colitis, also the newly discovered regulatory lymphoid cells (ILCreg) seem to be of pivotal importance^61^. These cells reside in the lamina propria of the murine and human intestine and expand during colitis. ILCreg, once activated, are a potent source of IL-10 and TGF-beta. In contrast to T cells, ILCreg effectively block IFNγ and IL-17 production, while not affecting IL-22, which is important for maintaining intestinal barrier function^62^.

IL-22 is an important cytokine for the resolution of colitis, since continuous microbial challenge in the context of impaired barrier function may inhibit resolution in colitis^63,64^. IL-22, which is produced by ILC3 activates epithelial stem cells, induces mucosal repair and thereby prevents the invasion of gut microbiota^62^ and the colonization with pathogenic bacteria such as segmented filamentous bacteria in colitis^62^. In the same line, IL-28 and IL-36 have shown to promote resolution of colitis by healing of epithelial damage and closing mucosal wounds. Activation of these cytokines also exerts permissive effects of IL-22 fostering repair responses and promoting the resolution of inflammation^65,66^. Modulation of the microbiota has been recently studied as novel approach for achieving resolution of colitis. Indeed, a pilot study using multi-donor stool transplantation led to promising clinical results in ulcerative colitis, suggesting that modulation of the microbiota may be targeted for resolution of inflammation^67^. Notably, in other organs such as the joints, which are not exposed to microbial load, ILC3 have a different role and act in a pro-inflammatory rather than resolving mode^45^.

Another, recently identified key resolution process in colitis is the activation of Tet methylcytosine dioxygenase 2 (TET2), which catalyzes the conversion of methylcytosine to 5-hydroxymethylcytosine^68^. Deficiency of Tet2 leads to long-lasting colitis without signs of resolution. Tet2 induces the resolution of arthritis by interaction with HDAC2 and repression of proinflammatory IL-6 gene locus by deacetylation.

### Resolution of lung inflammation

 The lungs are designed for effective clearance of infectious agents and other danger signals equipped with a high capacity for resolution of inflammation. Resolution of acute lung injury has shown to depend on the death and clearance of neutrophils^69^, IL-4/IL-13-mediated macrophage reprogramming^70^ and ILC2-mediated repair of lung tissue by amphiregulin, a member of the epithelial growth factor protein family that initiates epithelial repair after inflammatory damage^71^. Notably, some bacteria affecting the lungs, such as *Pseudomonas aeruginosa*, can disrupt resolution pathways and promote chronicity of inflammation, e.g., by expressing the hydrolase Cif, which cleaves and degrades pro-resolving lipids^72^. Also, during resolution of bacterial lung injury, a prolonged state of local immunosuppression can remain, which is based on TGF-beta-induced regulatory T cell accumulation^73^.

In contrast to acute lung injury and many other forms of inflammatory diseases, the process leading to chronicity of allergic airway disease depends on FoxO1-IRF4-mediated alternative macrophages activation^74^ and activation of ILC2s^75^. Hence, cells and their dependent cytokines, such as IL4, IL-5, and IL-13, which are normally act as pro-resolving mediators are hijacked in asthma to trigger inflammation. On the other hand, IL-10 is considered being important in the resolution of chronic lung inflammation. Hence, cardiolipin, a damage-associated molecular pattern, can inhibit the resolution of lung inflammation by repressing the IL-10 promoter through histone deacetylation by HDAC3^76^. Resolution of inflammation in the lung also requires the replacement of damaged type I alveolar cells, which is mediated by HIF-1a-induced proliferation and differentiation of type II alveolar cells. This process depends on activation of the beta-catenin pathway—an essential pathway for epithelial regeneration^77–79^. Asthma has a high potential to recur after resolution of inflammation, which can happen in conjunction with exogenous triggers or spontaneously. This recurrence of inflammation after resolution can be at least in part explained by allergen-experienced ILC2, which maintain memory-like properties and allow them to quickly respond after the resolution of inflammation^80^.

Natural killer (NK) cell have also been proposed to play a role in the resolution of allergic airway disease^81^. NK cells are diminished in their numbers in asthma and have lost essential cytotoxic properties, which may explain the increased number of leukocytes in the airways of asthma patients^82^.

Taken together, these data suggest that resolution of inflammation is orchestrated in a tissue-specific and disease-specific manner. The mechanisms that guide resolution in inflammatory processes do not seem to be uniform. In contrast, they appear to be tailored to effectively switch off the dominant cellular and molecular immune process that is also responsible for the activation of inflammation in the respective disease. This concept bears great advantages for the organism, as it can effectively stop inflammation and instigate tissue repair without the need to create a state of systemic immune suppression. It also demonstrates that resolution of inflammation should be perceived as a local process that aims to contain the immuno-inflammatory pathways operating in the affected tissues and organs, while not triggering a systemic change of the immune status of the individual. Furthermore, these findings suggest that the best strategies to therapeutically foster resolution of inflammation may vary from disease to disease and rather depend on effectively influencing the local inflammatory micro-milieu than inducing a state of systemic immune suppression. In the following chapter, potential therapeutic approaches that have the potential to strengthen resolution of inflammation will be discussed.

## Fostering resolution as a treatment option

Therapeutic induction of resolution is an attractive concept, but still in its infancy. To date, chronic inflammatory diseases, which are based on the failure of physiological resolution, are targeted via the suppression of effector pathways rather than stimulating resolution processes^3^. Current therapeutic approaches in arthritis, colitis, psoriasis, and asthma are directed against proinflammatory effector cytokines such as TNF-α, IL-6, IL-1, IL17, IL-12/IL-23, IL-5, or IL-4/13 using monoclonal antibodies or drugs against proinflammatory cytokine signaling pathways like Janus kinases^3,4,83^. Such approaches, although effective, bear the risk to blunt physiological immune responses and are therefore sometimes complicated by increased infection risk. Furthermore, such treatments usually have to be given life-long as they do not directly tackle the impaired resolution process.

Some newer treatment options, which relate to the regulation of immune cell influx, like the targeting the alpha4/beta7 integrin, or stimulate immune cell efflux, like inhibition of S1P1, may be closer to influencing resolution of inflammation than those tackling effector cytokines^84,85^. Fostering resolution may move treatments closer to cure by shutting down the pathological process rather than suppressing the inflammatory response^86^. Furthermore, such approach may also promote tissue repair. In consequence, pro-resolving therapies may also be rather safe and not complicated by higher infection risk. Whether such concept, however, is true in the clinic has not been determined to date.

Surprisingly, in some of the existing anti-inflammatory treatments pro-resolving functions appear to be embedded. For instance, glucocorticoids inhibit 5-ALOX, the key enzyme of leukotriene production, which may shift lipid synthesis to a more pro-resolving pattern^87^. Furthermore, glucocorticoids stimulate annexin 1 expression, in part through the glucocorticoid-induced leucine zipper, and thus induce efferocytosis^88^. Also, treatments such methotrexate inducing adenosine, as well as phosphodiesterase inhibitors like apremilast inducing cAMP levels and IL-10 production may at least in part act as inducers of resolution. However, such approaches do not specifically induce resolution.

More specific approaches to directly strengthen resolution may arise from the new insights in the metabolic control of macrophage function. Shifting macrophage metabolism from glycolysis to oxidative phosphorylation and changing the balance between citrate/succinate and itaconate in favor of the latter metabolite may not only effectively stop inflammation but also enhance resolution^89^. The principle, that targeting macrophage metabolism can entail anti-inflammatory effects, is supported by the anti-inflammatory function of the established anti-diabetic drug metformin: metformin inhibits ROS production from the electron transport chain and downregulates IL‐1β production, while boosting IL‐10 production^90^. In accordance, anti-inflammatory effects have been described for metformin in humans, which are independent from its function to control blood sugar concentrations^91^.

A number of additional therapeutic targets can be envisioned that modulate macrophage metabolism and may therefore support the induction of resolution of inflammation. For instance, molecules that activate AMP-activated protein kinase (AMPK), which is a key sensor of energy status of the cell and essential for the induction of oxidative phosphorylation, have shown to foster resolution of inflammation^92^. Itaconate itself may represent a pro-resolving agent as well and cell-permeable modifications of itaconate have been synthesized that are potential tools for inducing resolution of inflammation. Moreover, concepts mimicking the down-stream effects of itaconate, as an inducer of Nrf2 transcription factor expression, have already been developed. Among them, oleanane triterpenoid compounds, such as bardoxolone, are the most advanced drugs, which activate Nrf2, but have so far only been tested in diabetes mellitus and chronic kidney disease^93^. Anti-inflammatory effects in experimental arthritis have also been documented for compounds that block the succinate receptor GPR91^94^ as well as those blocking glycolysis, such as 3PO^95^, which fit well in the concept that the proinflammatory state of macrophages is associated with glycolysis (Warburg effect) and the accumulation of succinate. Furthermore, other approaches interfering with glycolysis, such as targeting pyruvate kinase M2, which is critical checkpoint for the Warburg effect, have been undertaken and have shown effective regulation of the expression of pro-inflammatory cytokines, such as IL-1β, in macrophages^96^.

Apart from modulating immune metabolic pathways and macrophage function, an interesting approach to enhance resolution of inflammation is of course the use pro-resolving lipid mediators, which preferentially target neutrophil influx. Such approaches have been reviewed elsewhere^6^. For instance, therapeutic use of resolvin E2 in mice has shown to promote resolution of inflammation, including shortening inflammation in experimental colitis^97^. Finally, a non-pharmacological approach to therapeutically affect resolution of inflammation is to make use of the efferent vagal nerve control of inflammatory responses^98^. This efferent branch of the neuro-immune reflex arch acts through acetylcholine and the engagement of a7 receptors on macrophages blocking their production of pro-inflammatory cytokines. In accordance, vagal stimulation blocks inflammatory cytokine production and promotes the resolution of arthritis, while vagotomy prolongs the resolution of inflammation^99^. First studies that used vagal stimulation by an implanted “pacemaker” to control inflammation have been accomplished with promising results in diseases such as arthritis^100^.

## Conclusions

In summary, resolution of inflammation is a tightly regulated process that restores tissue homeostasis and prevents the development of chronic inflammatory disease. Like the induction of inflammation, resolution is based on a network of cells as well as on lipid and protein mediators that cooperate for a common goal, which is to reconstruct the original state of the affected tissue. Apart from universal mechanisms tissue and disease-specific mechanisms have been identified that guide resolution of inflammation in arthritis, colitis, and asthma. Such pathways are of particular therapeutic value as their induction may provide new possibilities to reverse chronicity in distinct inflammatory diseases without curtailing the overall inflammatory response.
